# The effect of platform switching on the levels of metal ion release from different implant–abutment couples

**DOI:** 10.1038/ijos.2016.5

**Published:** 2016-05-27

**Authors:** Ghada O Alrabeah, Jonathan C Knowles, Haralampos Petridis

**Affiliations:** 1 Division of Biomaterials and Tissue Engineering, UCL Eastman Dental Institute, University College London, London, UK; 2 Prosthodontic Unit, Department of Restorative Dentistry, UCL Eastman Dental Institute, University College London, London, UK

**Keywords:** corrosion, dental implants, ion release, peri-implant bone loss, platform-switching, titanium

## Abstract

The improved peri-implant bone response demonstrated by platform switching may be the result of reduced amounts of metal ions released to the surrounding tissues. The aim of this study was to compare the levels of metal ions released from platform-matched and platform-switched implant–abutment couples as a result of accelerated corrosion. Thirty-six titanium alloy (Ti-6Al-4V) and cobalt–chrome alloy abutments were coupled with titanium cylinders forming either platform-switched or platform-matched groups (*n*=6). In addition, 18 unconnected samples served as controls. The specimens were subjected to accelerated corrosion by static immersion in 1% lactic acid for 1 week. The amount of metal ions ion of each test tube was measured using inductively coupled plasma mass spectrometry. Scanning electron microscope (SEM) images and energy dispersive spectroscopy X-ray analyses were performed pre- and post-immersion to assess corrosion at the interface. The platform-matched groups demonstrated higher ion release for vanadium, aluminium, cobalt, chrome, and molybdenum compared with the platform-switched groups (*P*<0.05). Titanium was the highest element to be released regardless of abutment size or connection (*P*<0.05). SEM images showed pitting corrosion prominent on the outer borders of the implant and abutment platform surfaces. In conclusion, implant–abutment couples underwent an active corrosion process resulting in metal ions release into the surrounding environment. The highest amount of metal ions released was recorded for the platform-matched groups, suggesting that platform-switching concept has a positive effect in reducing the levels of metal ion release from the implant–abutment couples.

## Introduction

Peri-implant alveolar bone loss has been established as one of the main criteria of implant success.^1^ This criterion requires <1.5 mm of crestal bone loss 1 year after abutment connection and <0.2 mm per year in the subsequent years.^1, 2^ Efforts have been directed to minimise the marginal bone resorption through different approaches. One of the approaches that demonstrated positive radiographic findings in respect to crestal bone level is the utilisation of the “platform-switched abutment” concept.^3^ “Platform switching is defined as a protocol that includes smaller diameter restorative components that have been placed onto larger diameter implant restorative platforms—the outer edge of the implant–abutment interface is horizontally repositioned inwardly and away from the outer edge of the implant platform”.^3^ Buser and colleagues observed a mean crestal bone loss for platform switching of 0.18 mm compared with 2.18 mm when standard platform-matched abutments were used.^4^ These positive findings of platform switching were supported in other clinical studies performed by different investigators, who also demonstrated that the positive results were also proportional to the amount of platform mismatch.^5, 6, 7, 8^ Several theories have been advocated to explain the concept of platform switching,^9^ including the biomechanical stress theory,^10, 11, 12^ the bacterial theory,^13, 14, 15^ and the biologic width theory.^3, 14, 16^ These theories have not totally succeeded in clarifying the exact mechanisms of peri-implant bone loss.^17, 18, 19, 20^ Therefore, the exact aetiology and mechanism behind its success could not be confirmed.

Bone remodelling around dental implants could be analysed by studying the orthopaedic literature that sheds light on the possible role of corrosion by-products and wear debris that concentrate in the surrounding peri-implant tissues.^21, 22, 23^ Such products include metal ions/particles derived from the materials used for the prosthetic treatment. As a result of the presence of particulate wear debris or corrosion products, a foreign body reaction could be initiated, leading to the development of osteolysis.^24, 25, 26, 27^ A possible reason for this phenomenon is that debris in tissues can influence various metabolic pathways, leading to cytokine release and interference of function of various cells such as osteoblasts, macrophages, lymphocytes, and fibroblasts, thereby disrupting bone homeostasis and contributing to the development of osteolysis.^25, 26, 27, 28, 29, 30, 31, 32, 33^

Metals are used for dental and orthopaedic implants because of their excellent mechanical properties, such as weight-to-strength ratio and good biological performance.^34, 35^ However, metallic devices are susceptible to corrosion, especially in challenging environments such as the oral cavity. In a highly corrosive environment such as the mouth, ion leakage from dental appliances occurs through different processes. Corrosion of dental implants and implants' superstructures may occur as a result of mechanical or electrochemical means^36^ such as crevice, pitting, and galvanic corrosion. Geis-Gerstorfer and colleagues stated that “the galvanic corrosion of implant/superstructure systems is important in the following two aspects: (1) the possibility of biological effects that may result from the dissolution of alloy components; and (2) the current flow that results from galvanic corrosion may lead to bone destruction”.^37^

Although metal ion release of elements, such as titanium and other metals, from dental implant prostheses might be small compared with the daily dietary intake of these elements,^38, 39^ corrosion cannot be ruled out when studying toxicity and hypersensitivity,^40, 41, 42, 43, 44^ and can have significant effects in a local environment. Therefore, the type of the released elements, the concentration, and the duration of exposure are factors that may affect the biological response.

On the basis of the aforementioned findings, it could be proposed that the release of corrosion by-products from the implant–abutment complex may contribute to the disruption of bone metabolism around dental implants. The improved peri-implant bone response demonstrated by various implant–abutment connection geometries, such as platform switching, may be the result of reduced amounts of metal ions released to the surrounding tissues. Therefore, the aim of this study was to compare the amount of metal ions released from platform-matched and platform-switched implant abutment couples as a result of accelerated corrosion.

## Materials and methods

### Preparation of test specimens

**Figure 1:**
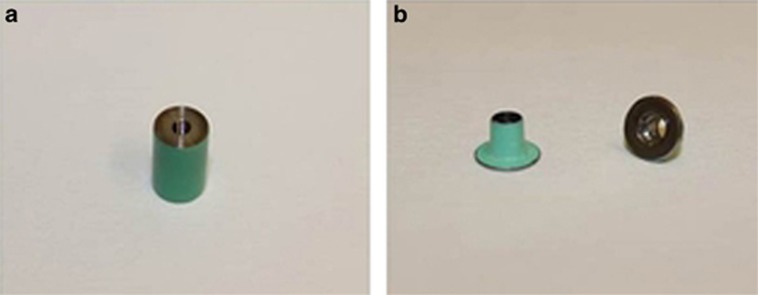
**Coated implant and abutment samples.** (**a**) Implant; (**b**) abutment.

Forty-two commercially pure titanium (CPTi) cylinders were fabricated to serve as dental implants (36 were connected to abutments and 6 remained unconnected). These implants were prepared by sectioning the as-received 6-mm diameter machined titanium rods (Medical grade titanium; Grade II, ASTM F67-13; Fort Wayne Metals, County Mayo, Ireland), into smaller cylinders each measuring 6 mm × 10 mm. Each cylinder was further machined to create a screw hole with screw threads tapped on one end producing an implant platform surface with a diameter of 6 mm (Figure 1a).

The abutments used were divided into two main groups according to their material. The first group (T) consisted of 24 prefabricated titanium alloy abutments (Ti-6Al-4V; Grade 5 Ti Alloy; Zfx-GmbH; Zimmer Dental, Dachau, Germany) with 18 abutments to be connected to implants and 6 abutments to remain unconnected. The connected samples of this is group (T) were further divided into three subgroups according to the abutment's platform diameter. Three platform diameters were selected for the connected samples; 6, 5 and 4 mm with six samples in each group. The 6-mm platform diameter represented the platform-matched group (M), whereas the 5-mm (SW) and 4-mm (S) diameter represented the platform-switched groups with two different amounts of mismatch between the implant and abutments. The unconnected abutments (UT) had a 4-mm platform diameter (*n*=6). The second abutment group (C) consisted of 24 cobalt–chrome (CoCr) abutments (18 to be connected to implants and 6 to remain unconnected) and were produced using computer assisted design/computer assisted manufacturing (CAD/CAM) technology and a “laser melting” process (LaserAbutments; Renishaw, Wotton-under-Edge, UK). These abutments were designed by scanning of three titanium abutments from the first group, which all measured 4 mm in height but differed in their platform diameter (6, 5, and 4 mm). Therefore, the connected samples of the second group (C) were also divided into three subgroups according to the abutment's platform diameter similar to that of the titanium group. The unconnected CoCr abutments (UC) had a 4-mm platform diameter (*n*=6). The platform surface of all the abutments and implants was subjected to wet polishing on a 4 000-grit silicon carbide polishing discs (LaboPol-5; Stuers, Copenhagen, Denmark). Following polishing, all the implants and abutment specimens were separately cleaned by rinsing for 2 min in 70% ethanol in an ultrasonicator (Branson 5800; Branson Ultrasonics, Danbury, CT, USA). The specimens were then rinsed in deionized water and dried with oil- and water-free compressed air according to ISO 10271.^45^ All the surfaces of the implants and abutments were coated with commercial resin except for the contacting surfaces of the platform to limit the corrosion effect to the interfacial area only (Figure 1a and 1b).

The composition of the implant and abutment materials used in this study is presented in Table 1.

### Immersion protocol

**Figure 2:**
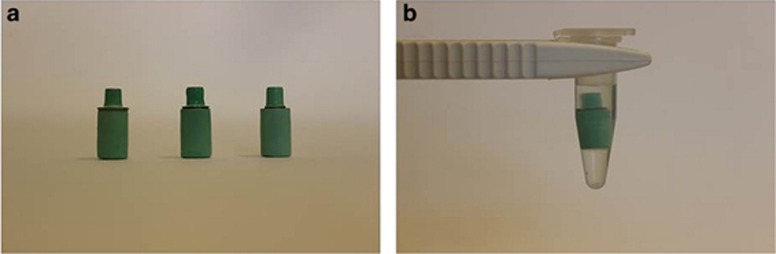
**Implant–abutment couples**. (**a**) Implant-abutment couples with three abutment sizes; (**b**) an implant–abutment couple sample statically immersed in 1% lactic acid solution for 1 week at 37 °C.

Before exposure to the immersion solution, 36 implants were connected to their assigned abutments using hexed titanium screws, and tightened manually (Figure 2a). The remaining six implants as well as the remaining six Ti abutments and six CoCr abutments remained unconnected. A total of 54 specimens (*n*=6) formed nine groups (Table 2).

Fresh 1% lactic acid aqueous solution (comprising 0.1 mol·L^−1^ lactic acid and 0.1 mol·L^−1^ sodium chloride) was prepared immediately before use (pH=2.3) according to ISO 10271. A volume sufficient to produce a ratio of 1 mL of solution per cm^2^ of sample surface area was added to all test tubes.^45^ Specimens were statically immersed and they were completely covered by lactic acid (Figure 2b). All test tubes were covered with lids to prevent evaporation and were maintained in an incubator at 37 °C for 1 week under static conditions in accordance with ISO 10271.^45^

An additional test tube with a completely coated titanium cylinder immersed in 1 mL of test solution was used as a reference and was maintained in parallel with the solutions containing the specimens. This reference solution was used to establish the impurity level for each element of interest in the lactic acid solution. After 7 days, extracts of immersion solution were collected from each test tube, including reference solution, dissolved in 2% nitric acid (HNO_3_), and stored under refrigeration (4 °C) until required for elemental analysis.

### Quantification of metal ion release

The quantification of the metal ions released from the implant–abutment couples into the immersion solutions was carried out using inductively coupled plasma mass spectrometry (ICP-MS; Varian/Bruker 800-MS Series; Analytical West, Corona, CA, USA). Elements analysed were titanium (Ti), vanadium (V), aluminium (Al), cobalt (Co), chromium (Cr), and molybdenum (Mo). The results were presented as part per billion (ppb, × 10^−9^).

### Observation of the contacting surfaces of the implant and abutment before and after immersion

Before connecting the abutments to the implants, two representative specimens from each subgroup of the abutments (total 12) and their corresponding 12 implants were randomly selected for examination of the contacting platform surfaces under scanning electron microscope (SEM; FEI, Eindhoven, Netherlands). Energy dispersive spectroscopy X-ray (EDX; Inca 400 EDX; Oxford Instruments Analytical, High Wycombe, UK) analyses were also performed on five spot areas of each contacting surface to assess the elemental composition.

After the immersion test, the same two representative specimens that were examined before immersion test were disconnected, cleaned, and examined again under SEM and EDX to assess corrosion at the interface.

### Statistical analysis

Levene's test was employed to test for homogeneity. When the distribution was homogenous, one-way analysis of variance was used, followed by *post hoc* multiple comparisons test applying a Bonferroni correction to find patterns between the subgroups. The Kruskal–Wallis test was employed when the distribution was not homogenous. Mann–Whitney's test was used for detecting differences between the connected and unconnected groups. The significance level was set at 5%.

## Results

### Metal ion release

Ti release was the highest among all the elements tested (*P*<0.05) ranging from 440 × 10^−9^ for the UT group to 1 250 × 10^−9^ for the TM group, regardless of abutment size, abutment material, or whether samples were connected or not.

#### Comparison within the connected groups

**Figure 3:**
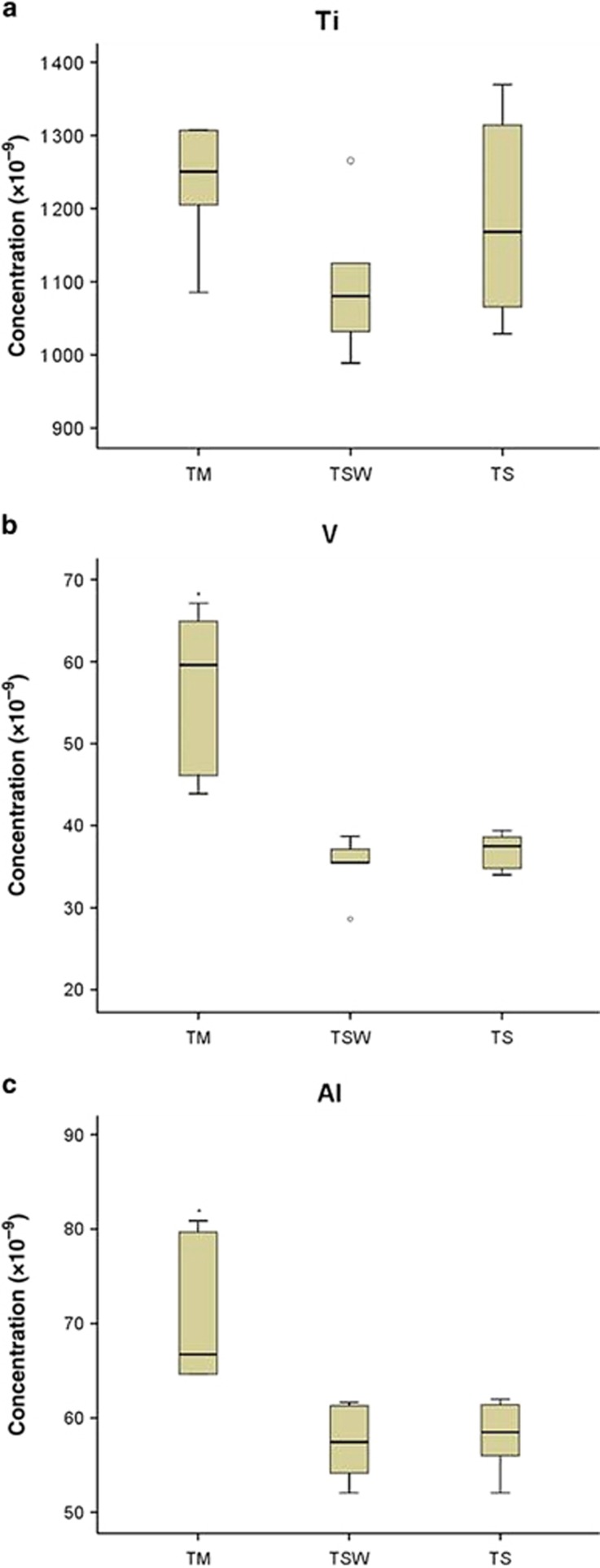
**Metal ion release from Ti alloy abutment group.** Ion release for titanium (Ti), vanadium (V), and aluminium (Al) elements from implants connected to titanium alloy abutments (Ti-6Al-4V) after static immersion for 1 week in 1% lactic acid solution. The results are expressed as median concentrations in parts per billion (ppb, × 10^−9^) and quartiles. *n*=6 per group (*P<0.05). TM, connected platform-matched titanium abutment (6 mm); TSW, connected platform-switched titanium abutment (5 mm); TS, connected platform-switched titanium abutment (4 mm).

The highest release of Ti was recorded for the implant connected to platform-matched Ti abutment group (TM=1 250 × 10^−9^), but no statistically significant differences (*P*>0.05) between the platform-matched and platform-switched groups within the Ti abutment material group were detected (Figure 3a). Vanadium demonstrated the highest release in the implant connected to the platform-matched Ti abutment group (TM=60 × 10^−9^), which was statistically significantly higher than the other two platform-switched groups (TSW=36 × 10^−9^, TS=38 × 10^−9^; *P*<0.05; Figure 3b). Similarly, Al release was statistically significantly higher in the TM group (67 × 10^−9^) than the other two platform-switched groups (TSW=57 × 10^−9^, TS=59 × 10^−9^; *P*<0.05; Figure 3c).

**Figure 4:**
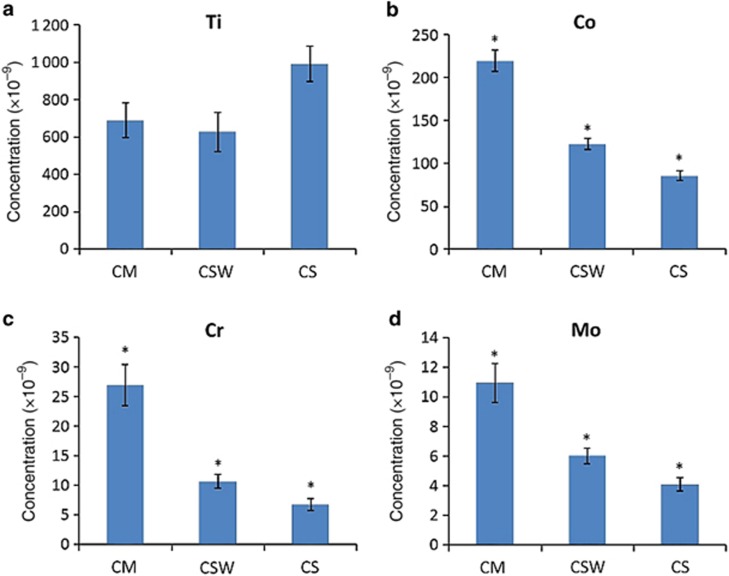
**Metal ion release from the CoCr abutment group.** Ion release for titanium (Ti), cobalt (Co), chromium (Cr), and molybdenum (Mo) from implants connected to cobalt–chrome abutments after static immersion for 1 week in 1% lactic acid solution. The results are expressed as mean concentration in parts per billion (ppb, × 10^−9^)±standard deviation *n*=6 per group (*P<0.05). CM, connected platform-matched cobalt–chrome abutment (6 mm); CSW, connected platform-switched cobalt–chrome abutment (5 mm); CS, connected platform-switched cobalt–chrome abutment (4 mm).

On the other hand, the release of Ti from the implants connected to the CoCr abutments group was higher in the CS group (993 × 10^−9^); however, it was not significantly different than the platform-matched group (CM; *P*>0.05), but was significantly higher than the CSW group (*P*<0.05; Figure 4a). Co was the second highest element to be released with its highest release being in the implant connected to the platform-matched cobalt–chrome abutment group (CM=219 × 10^−9^; *P*<0.05). The release of Co significantly decreased as the size of the abutment decreased in the connected samples (*P*<0.05) demonstrating its lowest release in the implant connected to the platform-switched cobalt–chrome abutment group (CS=85 × 10^−9^; Figure 4b). Similarly, Cr and Mo had the same tendency as Co where their highest release was observed in the implant connected to the platform-matched cobalt–chrome abutment group (CM) and as the size of the cobalt–chrome abutment decreased, the Cr and Mo leakages also decreased significantly (*P*<0.05; Figure 4c and 4d).

#### Comparison between the connected and unconnected groups

The UI showed significant higher release of Ti (UI=998 × 10^−9^) when compared with the connected implants in the CM and CSW groups (*P*<0.05); however, no differences between the release of titanium from the UI and the CS group (*P*>0.05).

When comparing the release of Co, Cr, and Mo from the UC group to that of the CS, it was found that the release of Co was significantly higher in the UC group (103 × 10^−9^; *P*<0.05). However, there was no significant difference in Cr and Mo release between the UC and CS groups (*P*>0.05).

The release of V and Al was significantly lower in the UT (V=17 × 10^−9^, Al=10 × 10^−9^) compared with its release from the TS group (*P*<0.05).

### Pre- and post-immersion SEM

**Figure 5:**
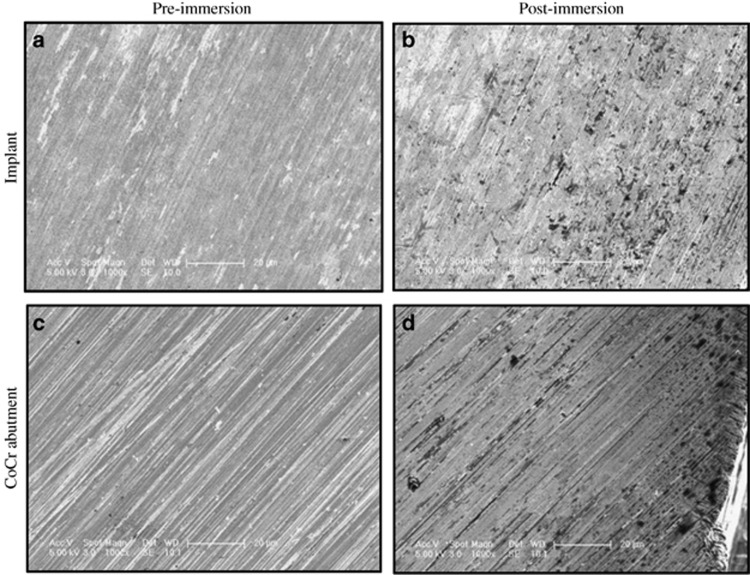
**SEM images of the contacting surfaces of an implant and a CoCr abutment.** Magnification x1 000. A titanium cylinder and a CoCr abutment from the CSW group before and after immersion for 1 week at 37 °C in 1% lactic acid solution. CoCr, cobalt–chrome; CSW, connected platform-switched cobalt–chrome abutment (5 mm); SEM, scanning electron microscopy.

**Figure 6:**
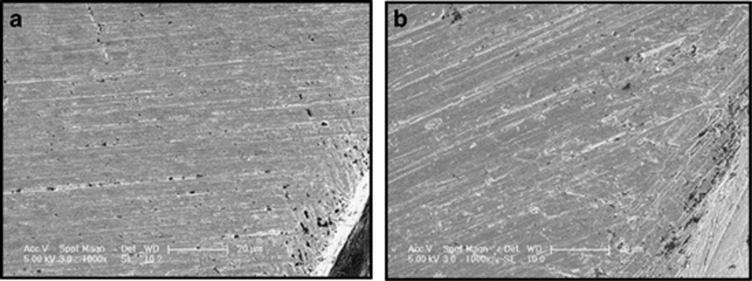
**Post-immersion SEM images of the contacting surfaces of an implant and a Ti alloy abutment.** (**a**) Implant; (**b**) Ti abutment. Magnification x1 000. Pitting corrosion at the outer border of a titanium implant and a Ti alloy abutment from the TM group after immersion for 1 week at 37° in 1% lactic acid solution. SEM, scanning electron microscopy; Ti, titanium; TM, connected platform-matched titanium abutment (6 mm).

Post-immersion SEM images showed active corrosion process demonstrated as pitting areas on the interfacial surfaces of both the implants and their opposing abutments in all test groups when compared with the pre-immersion images (Figure 5). The pitting areas, however, were more pronounced closer to the outer borders of the implants and abutment surfaces (Figure 6).

### Pre- and post-immersion EDX analysis

**Figure 7:**
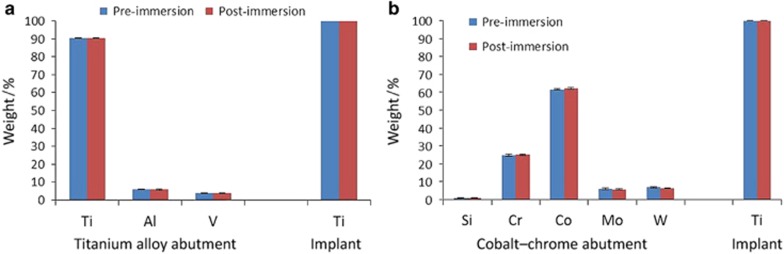
**Pre- and post-immersion EDX elemental analysis of the contacting surfaces of implants and their opposing abutments in weight.** Elemental analysis of an implant connected to a platform-matched titanium abutment and an implant connected to a platform-switched cobalt–chrome abutment before and after immersion for 1 week at 37° in 1% lactic acid solution. (**a**) Titanium abutment; (**b**) Cobalt–chrome abutment. The results are expressed as mean composition in weight±standard deviation. *n*=2. Al, aluminium; Cr, chromium; Co, cobalt; EDX, energy dispersive spectroscopy X-ray; Mo, molybdenum; Si, silicon; Ti, titanium; V, vanadium; W, tungsten.

EDX analysis of the interfacial contacting surfaces of the implants showed the presence of mainly Ti element (>99.9%). No elements from the abutment materials were deposited on the implants of all test groups, whether connected to Ti or CoCr abutments of different sizes, after 7 days of immersion in lactic acid, when compared with pre-immersion analyses. Similarly, there were no major differences in the element content between the pre- and post-EDX analysis of the surfaces of the Ti and CoCr abutments of all three sizes (Figure 7a and 7b).

## Discussion

In the present study, corrosion of titanium implants connected to Ti and CoCr abutments with different diameters, representing the platform-switched and platform-matched groups, was evaluated by static immersion tests,^45^ and analysed by ICP-MS in order to quantify the amount of released elements. Other corrosion tests could include electrochemical testing using potentiodynamic polarisation. However, assessment of electrical potential was not performed in this study because when investigating the biologic effects of corrosion products; element leakage measurement is considered appropriate.^46^

Two abutment materials were used in this study; a titanium alloy (Ti-6Al-4V) and a cobalt–chrome metal alloy. Having different superstructure material would have an influence on the galvanic corrosion phenomena. Titanium behaves differently when connected to different materials; it acts as an anode when connected to a noble metal such as gold, whereas it is considered the cathode when connected to a base metal. Therefore, assessing the effect of platform-switching (having smaller abutment diameter) necessitates using different materials on top of the titanium implant platform because one of the critical basic rules of corrosion science is that galvanic corrosion is inversely related to the surface of the anode, which means that corrosion decreases when the anode surface is larger than the cathode.^47, 48^ In the present test, gold was not used as an abutment material and the CPTi implants represented the cathodic region of the galvanic cells. It would be interesting to see the effect of gold alloy abutments in future studies.

The high variations in elements leached, observed in the ICP-MS results, especially for the Ti element is related to the sensitivity of such analytic method. Further limitations are caused by sample preparation, instrument resolution and detection limits, challenging interferences during measuring processes and examiner's experience. However, it was still clear from the ICP-MS results that the platform-matched group within each abutment material demonstrated relatively higher element leakage compared with the other platform-switched groups (approximately twofold higher for V, Co, Cr, and Mo). These differences in element release were statistically significant for V, Al, Co, Mo, and Cr. An initial explanation for these results might be the different surface areas that are in contact, as this has been shown to influence galvanic corrosion. As this experiment was a static corrosion test, the amount of corrosion products would be expected to increase if more modes of corrosion were introduced, such as fretting corrosion. To the authors' knowledge, there is no published study that has looked into elemental leakage from implant–abutment couples with different connection geometries, and hence the results of this study are not directly comparable to other studies with concern to the effect of abutment size and amount of platform mismatch.

Although Ti is a material with a high corrosion resistance compared with other metallic materials used in oral rehabilitation,^49^ it demonstrated the highest leakage among all the elements tested in this study. This finding is in agreement with the results obtained by Okazaki and Gotoh^50^ in 2005 where they showed that Ti release in lactic acid solution (pH=2.6) was higher than the release of Co, Cr, Mo, V, and Al in the same solution.^50^ These authors also demonstrated that the release of Ti in lactic acid was higher than its release in all the other physiologic solutions tested. Similarly, Koike and Fuji^51^ showed that the release of Ti was high in lactic acid (pH=2.5) and its release increased as the pH level decreased.^51^ This behaviour of Ti is probably due to the dissolution of the protective titanium oxide (TiO_2_) film in aggressive environmental conditions, with high concentration of electrolytes and low pH, such as those experienced in the present study. Therefore, for *in vitro* tests assessing the corrosion behaviour of dental devices, the selection of the test solution is extremely significant and the reproduction of the oral environment is desirable. Solutions simulating the biological conditions include 0.9% NaCl solution, phosphate-buffered saline, and artificial saliva.^52, 53^ Even though body fluids are buffered, pH variations are possible such as during inflammation.^54^ In addition, dental implants are often associated with dental plaque in which the pH drops to below 4.5 for extended time due to the presence of acidogenic bacteria in the oral cavity.^49, 51, 55^ In a study performed by Yoneyama *et al*.,^56^ they explained that 0.9% NaCl solution simulated the body fluid to a certain extent, but was deficient in quantitative features, specifically, the reliability of the released Ti ion levels, and 1.0% lactic acid solution was found to be more appropriate.^57^ A number of other corrosion studies have utilised lactic acid^37, 57, 58^ and it is the solution of choice according to the ISO 10271.^34^ Accordingly, 1.0% lactic acid solution with pH 2.3 was chosen for this test to accelerate the corrosion process.^45^

The ion release of Ti and Co from the unconnected samples was higher than their corresponding samples from the connected groups. Hjalmarsson *et al.*^59^ showed similar results in a study investigating material degradation from implant retained CoCr and Ti frameworks. The authors measured the release of Ti, Co, and Cr from connected and unconnected frameworks in artificial saliva and demonstrated higher metal ion release from the unconnected frameworks in all elements tested.^59^ This could be due to a wider surface area that is in direct contact with the electrolyte leading to electrochemical material dissolution. This finding indicated that even in the same environmental conditions, materials of the same design and composition may behave differently, with regard to their corrosion behaviour, depending on the mode in which they exist in such environment (connected or unconnected, loaded or unloaded, and so on). Accordingly, different modes of corrosion are anticipated leading to different amounts of corrosion products.

Corrosion results in the release of metallic ions into the surrounding tissues that can initiate and stimulate an initial inflammatory response, and a consequent toxic, mutagenic, and/or carcinogenic reaction.^49^ The concentration of metal ions or particles has been found to be directly proportional to the phagocytic response up to a saturation level.^60^ Sun *et al.*^61^ demonstrated that the effects of metal ions (Ni, Co, Ti, and V) on cell viability were a function of their concentrations.^61^ Some biological processes do not require large differences in metal ion concentrations to cause a change in the biological response. Zijlstra *et al.*,^62^ using more clinically relevant concentrations (1 × 10^−9^−100 × 10^−9^), which were also close to the concentrations found in the present study, recently showed that Co and Cr ions reduce the cell number, cell activity, and the expression of osteoprotegerin and receptor activator of nuclear factor kappa B ligand, which are significant for bone formation and resorption,^63^ in human osteoblasts with almost all the concentrations tested (1 × 10^−9^−100 × 10^−9^). The observed reduction was also dependant on the ion dosages of Co and Cr.^62^ The fact that differences in the amount of metal ions released have been detected in the present study between platform matched and platform switched suggests that such differences may lead to differing biological responses in the surrounding tissues. The platform-switched samples released relatively lower amounts of metal ions, with a statistically significant decrease for V, Al, Co, Cr, and Mo. Moreover, the decrease was proportional to the amount of mismatch for the Co, Cr, and Mo elements. Similar clinical results, regarding the effect of the amount of mismatch, have been recently published by Canullo *et al.*^64^ who reported that marginal bone loss was inversely related to the degree of platform switching after almost 3 years of observation.^64^

The SEM images in this study also showed that corrosion was more prominent at the outer borders of the contacting surfaces from which the solution leaks through the microgap into the interface. Therefore, it could be assumed that the closer the contacting surfaces are to the surrounding tissues, as in the platform-matched abutments, the closer the degradation products are able to reach the tissues and therefore the more biological effects are expected. In other words, as the implant abutment mismatch increases, the further the degradation products are from reaching the tissues, providing a possible explanation for the clinical results reported by Canullo *et al.*^64^ However, further *in vitro* and *in vivo* investigations are needed to validate such correlation, and therefore caution must be considered when drawing conclusions.

The SEM images showed active corrosion processes on the surfaces of both the Ti implants and abutments, and that corrosion was more prominent on the CoCr abutments regardless of their sizes. This finding is in agreement with the results of Tuna *et al.* who demonstrated that the CoCr superstructures were extremely prone to corrosion.^52^ However, they observed no significant change in the SEM images of the Ti implants before and after corrosion. This difference between the present study and the findings by Tuna with regards to Ti corrosion could be due the different electrolytic medium utilised. Tuna *et al.*^52^ immersed their coated samples in an Afnor-type^65^ artificial saliva buffered to pH 6.7. Another reason could be due to different modes of corrosion tests between the two studies and the use of different implant materials with different surface finish.^52^ In the present study, machined titanium cylinders were utilised to represent the actual implants and were constructed from CPTi grade II. Although this might be considered a limitation, titanium cylinders representing the implant fixtures are thought to be appropriate and have been used by several investigators when measuring metal ion leakage with no loading conditions.^57, 65, 66^

EDX analysis for elemental composition^65, 67, 68^ did not show the presence of Co, Cr, Mo, Al, or V on the examined areas of the implants nor the presence of Ti on the CoCr abutments after the accelerated corrosion procedure, in other words there was not any deposition of the abutment's elements on the implants' surface and vice versa. This finding supports the idea that corrosion products are completely released into the surrounding environment of the implant abutment couple and that such products gain access to the peri-implant tissues through the microgap. The presence of corrosion products in peri-implant tissues has been confirmed in histologic analysis of biopsies of peri-implantitis specimens.^68^ The histopathologic findings demonstrated the presence of foreign bodies surrounded by chronic inflammatory infiltrates. The foreign bodies were of different elemental composition, however, titanium was predominant. The authors suggested that the appearance of titanium particles in the peri-implantitis biopsies could be due to implant corrosion.^68^ The results of the present study support this possibility^68^ and suggest that implant–abutment couples undergo active corrosion process in the oral environment that leads to the release of metal ions/particles to the peri-implant tissues. However, due to the extremely complex nature of interactions between dental biomaterials and the biological oral environment, complete understanding of the possible clinical consequences of the various amounts of corrosion elements released in this study is not possible. Therefore, further research is required looking into the effects of the various element concentrations on various metabolic pathways related to bone homeostasis.

Within the limitation of the current study, it could be concluded that the implant–abutment couples underwent an active corrosion process prominent on the outer borders of the contacting surfaces of the implants and abutments, resulting in metal degradation products completely released into the surrounding environment. The highest amount of metal ions released was recorded for the platform-matched groups. These findings suggest that platform-switching concept has a possible positive effect in reducing the levels of metal ion release through corrosion processes from the implant–abutment couples into the surrounding environment. On the basis of the literature, this reduction may partly explain the positive radiographic findings in respect to crestal bone level when utilising the “platform-switching” concept, providing, for the first time, some indirect evidence for the possible role of corrosion products in the mediation of crestal bone loss around dental implants. Further studies are already being conducted by the current research group, looking into the effect of such differences on various cell metabolic pathways.

## Figures and Tables

**Table 1:** Chemical composition of the alloys in weight (%) according to the respective manufacturer

Material	Element													
	Co	Cr	Mo	W	Mn	Si	Ti	V	lA	Fe	N	C	O	H
CPTi grade II							>99.5			0.2	0.03	0.1	0.18	0.015
CoCr	63.1	24.7	5.4	5.1	<1	1>				<1				
Ti-6Al-4V*							91	4	6					

CPTi, commercially pure titanium; CoCr, cobalt–chrome.

Chemical composition was obtained by elemental analysis using energy dispersive X-ray spectroscopy and was not provided by the manufacturer.

**Table 2:** Samples groups and their corresponding codes

Sample name	Code	Number of samples
Unconnected implant	UI	6
Unconnected titanium abutment (4 mm)	UT	6
Unconnected cobalt–chrome abutment (4 mm)	UC	6
Connected platform-matched titanium abutment (6 mm)	TM	6
Connected platform-switched titanium abutment (5 mm)	TSW	6
Connected platform-switched titanium abutment (4 mm)	TS	6
Connected platform-matched cobalt–chrome abutment (6 mm)	CM	6
Connected platform-switched cobalt–chrome abutment (5 mm)	CSW	6
Connected platform-switched cobalt–chrome abutment (4 mm)	CS	6
Total		54
